# Identification of Novel Candidate Oncogenes in Chromosome Region 17p11.2-p12 in Human Osteosarcoma

**DOI:** 10.1371/journal.pone.0030907

**Published:** 2012-01-26

**Authors:** Joeri Both, Thijs Wu, Johannes Bras, Gerard R. Schaap, Frank Baas, Theo J. M. Hulsebos

**Affiliations:** 1 Department of Genome Analysis, Academic Medical Center, Amsterdam, The Netherlands; 2 Department of Pathology, Academic Medical Center, Amsterdam, The Netherlands; 3 Department of Orthopedic Surgery, Academic Medical Center, Amsterdam, The Netherlands; University of Massachusetts Medical School, United States of America

## Abstract

Osteosarcoma is the most common primary malignancy of bone. The tumours are characterized by high genomic instability, including the occurrence of multiple regions of amplifications and deletions. Chromosome region 17p11.2–p12 is amplified in about 25% of cases. In previous studies, *COPS3* and *PMP22* have been identified as candidate oncogenes in this region. Considering the complexity and variation of the amplification profiles for this segment, the involvement of additional causative oncogenes is to be expected. The aim of the present investigation is to identify novel candidate oncogenes in 17p11.2–p12. We selected 26 of in total 85 osteosarcoma samples (31%) with amplification events in 17p11.2–p12, using quantitative PCR for 8 marker genes. These were subjected to high-resolution SNP array analysis and subsequent GISTIC analysis to identify the most significantly amplified regions. Two major amplification peaks were found in the 17p11.2–p12 region. Overexpression as a consequence of gene amplification is a major mechanism for oncogene activation in tumours. Therefore, to identify the causative oncogenes, we next determined expression levels of all genes within the two segments using expression array data that could be generated for 20 of the selected samples. We identified 11 genes that were overexpressed through amplification in at least 50% of cases. Nine of these, *c17orf39*, *RICH2*, *c17orf45*, *TOP3A*, *COPS3*, *SHMT1*, *PRPSAP2*, *PMP22*, and *RASD1*, demonstrated a significant association between copy number and expression level. We conclude that these genes, including *COPS3* and *PMP22*, are candidate oncogenes in 17p11.2–p12 of importance in osteosarcoma tumourigenesis.

## Introduction

Osteosarcoma is the most frequent primary bone tumour in children and adults with a peak incidence in adolescence and a second smaller peak after age 50 [1]. The tumours develop mainly in regions with high osteoblastic activity like the metaphyseal regions of the long bones, including the distal femur, proximal tibia and proximal humerus [2]. Primary osteosarcomas arise from primitive mesenchymal cells producing osteoid [3]. The main site of metastasis is the pulmonary region and the metastasized secondary tumours, present at presentation in 10–20% of patients, are often the cause of death [4]. The introduction of pre- and postsurgical chemotherapy has increased survival dramatically during the last three decades, reaching five-year survival rates of 60–75% for patients with localized disease [5], [6]. However, further improvements in survival rate are obviously needed. One way to accomplish this is the identification of molecular biomarkers for osteosarcoma, which may be used for the improvement of diagnosis and as possible targets for therapy.

Osteosarcomas are genetically characterized by high genomic instability, including a high degree of aneuploidy, the presence of unbalanced chromosomal rearrangements, and the occurrence of multiple amplified or deleted segments (reviewed by Sandberg and Bridge [7]). Despite this genomic complexity, chromosomal and array comparative genomic hybridization (CGH) studies have revealed a limited number of chromosomal regions that are consistently involved in high copy number gain or amplification events, including 6p12-p21, 8q24, and 17p11.2-p12 [8]–[10]. These regions are expected to contain one or more oncogenes of which the amplification-induced overexpression is important for osteosarcoma tumourigenesis. Indeed *RUNX2* and *CDC5L*, both encoding cell cycle regulators, in 6p12-p21, and *MYC* in 8q24 have already been identified as likely candidate oncogenes for osteosarcoma in these chromosomal segments [11]–[13]. In an earlier small-scale study, we found genes *COPS3* in 17p11.2 and *PMP22* in 17p12 to be most consistently overexpressed after amplification in osteosarcomas, making these genes candidate oncogenes in that segment [14]. However, due to technical restrictions, the expression status of only about 60 genes and expressed sequence tags in the amplified region could be analyzed. Moreover, the available (chromosome and array) CGH and microsatellite marker data from us and others did not allow an accurate demarcation of the amplified segments within 17p11.2-p12 in the analyzed tumours [8]–[10], [15]–[19].

Recent studies in other tumour types suggest that amplicons may contain multiple oncogenes that can act independently or cooperatively [20], [21]. In order to identify all possible candidate oncogenes in the 17p11.2-p12 region, we performed a detailed amplification and expression profiling of this segment in osteosarcomas. To increase the sensitivity of our assays, we restricted our analyses to tumours that were selected for the presence of amplification events in that region.

## Materials and Methods

### Osteosarcoma samples and osteoblasts

Eighty-five osteosarcomas were selected form a collection, which was established over a period of 15 years (1993–2008) at the Academic Medical Center in Amsterdam. Age of the patients (53% male/47% female) ranged from 6 to78 (median 16). For those in the 17p-selected group (40% males/60% females) age ranged from 6 to 58 (median 16). All osteosarcoma samples were checked for high (>90%) tumour cell content by an experienced pathologist (J.B.). Human primary fetal osteoblasts were cultured in osteoblast basal medium with osteoblast growth supplement (Cell Applications, Inc, San Diego, CA USA).

### Ethics statement

The research was performed at the Department of Genome Analysis of the Academic Medical Center (AMC), Amsterdam, The Netherlands. Clinical samples were irreversibly anonymised and results of scientific research could not be linked to individual patients. The Committee Medical Ethics of the Academic Medical Center specifically waived approval for this study because it falls under paragraph 7:467 Civil Law Code of The Netherlands.

### DNA and RNA extraction

Tumour tissue samples were cut in 10 µm sections. These were collected in alternate fashion for DNA and RNA isolation. Genomic DNA was isolated with proteinase K digestion and chloroform extraction according to standard methods. For RNA extraction, fetal osteoblasts were cultured to 90% confluency. Total RNA was extracted from tumour tissue sections and cultured osteoblasts using the Trizol method (Invitrogen, Breda, The Netherlands) and additionally purified according to the RNeasy protocol (Qiagen, Venlo, The Netherlands). RNA quality was assessed using the BioAnalyzer (Agilent Technologies Inc, Palo Alto, CA, USA). Only samples with RNA Integrity Number (RIN) score higher than 7.5 were used for subsequent analyses.

### Quantitative Real-Time PCR

For gene dosage measurements, quantitative PCR (qPCR) was performed using the LightCycler 480 Real-Time PCR system (Roche, Almere, The Netherlands) according to the manufacturer's instructions. Target gene dosages in the tumour tissue samples were normalized against reference gene *ALB*. Primers and probes for the target genes and reference gene were designed using the UniversalProbe Library Assay Design Center (Roche, http://www.roche-applied-science.com/sis/rtpcr/upl/ezhome.html) and are listed in Supplemental Table S1. Primers were tested *in-silico* for specificity (http://www.genome.ucsc.edu/cgi-bin/hgPcr) and on agarose gels for single band amplification. All PCR reactions were performed in triplicate. Normalized ratios were calculated using the Relative Quantification software, provided by the manufacturer, and normal blood as reference. A normalized ratio of 1 is equivalent to the presence of two copies of a target gene in the tissue under study. Amplification was defined as target gene copy number >3.5 in the tumour sample.

### Quantitative Real-Time RT-PCR

Quantitative reverse transcription (RT) PCR was used for measurement of mRNA expression levels of genes. For this purpose, total RNA (0.5 ug) was reverse transcribed into cDNA by oligo-dT or random priming, according to standard methods. The cDNA content of target genes in the tumour tissue samples was normalized against that of reference gene *SDHA*. Design of primers and probes, listed in Supplemental Table S2, specificity testing, and PCR reactions were performed as described for the qPCR method. Fold changes in the expression of target genes were calculated as described above, using cultured human fetal osteoblasts as reference. Target genes that showed a 2-fold or more increase in expression in the tumour sample compared to the expression in the osteoblasts sample were considered to be overexpressed.

### Single nucleotide polymorphism (SNP) array analysis

DNAs extracted from tumours with significant amplifications in 17p11.2-p12 were analyzed for whole-genome copy number variation by using Illumina HumanCNV370-Quad BeadChips. The arrays contain probes for 373,397 SNPs. Processing of DNA samples, hybridization, staining, and scanning of the BeadChips, and primary data extraction were all performed according to the Illumina Infinium II protocol at the array facility of ServiceXS (Leiden, the Netherlands). Scoring of genotypes was done using the standard cluster file from Illumina. The arrays were analyzed with Illumina GenomeStudio software (version 2009.2). The LogR ratio (LRR) and the B allele frequency (BAF) data were processed into the OverUnder plugin as described by Attiyeh *et al* [22], and copy numbers were calculated using Illumina BeadStudio (version 3.2.2). The OverUnder algorithm calculates copy numbers of tumours based on the allelic imbalance and signal intensities and corrects for aneuploidy. The resulting copy numbers calculated for each SNP were then used as input for Gain and Loss Analysis of DNA (GLAD) [23]. GLAD detects the altered regions in the genomic pattern and assigns labels of “normal”, “gained” or “lost” to the segmented data. These output data were used as input for Genomic Identification of Significant Targets in Cancer (GISTIC) analysis [24]. This program identifies and analyses significant chromosomal aberrations across a set of tumour samples, based on the amplitude of the aberrations as well as their frequency of occurrence across the samples and calculates a value for this significance, called the G-score. Multiple hypothesis testing was accounted for using False Discovery Rate (FDR) q-value statistics. A cutoff q-value of 0.25 was used to select significant regions. The SNP data are described in accordance with MIAME guidelines and have been deposited in the NCBI's GEO Omnibus database under accession number GSE32964.

### Expression microarray analysis

RNAs extracted from tumours with significant amplifications in 17p11.2-p12 and from human fetal osteoblasts were analyzed for whole genome gene expression analysis using Illumina HumanHT-12 v3 Expression Beadchips. Each array contained 48,804 probes, spanning the entire human transcriptome. Labeling of RNA samples, hybridization, staining, and scanning of the Beadchips, and primary data extraction were all performed according to the Illumina Infinium II protocol at the array facility of ServiceXS (Leiden, the Netherlands). Fold changes in expression of a probe were determined by normalizing the intensity of the average signal in the tumour sample against the average signal in the osteoblasts sample. The expression microarray data are described in accordance with MIAME guidelines and have been deposited in the NCBI's GEO Omnibus database under accession number GSE32964.

## Results

### SNP array analysis identifies two significant amplicons in 17p11.2-p12

**Figure 1:**
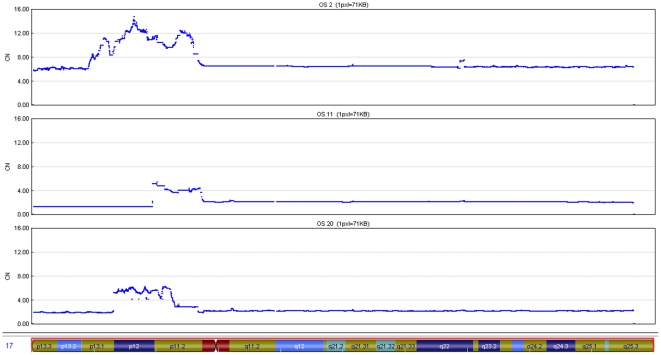
Representative examples of copy number (CN) changes along chromosome 17 in osteosarcomas OS2, OS11, and OS20. SNP-array data were processed using the OverUnder algorithm described by Attiyeh *et al* [*22*], and copy numbers were calculated using Illumina BeadStudio.

**Figure 2:**
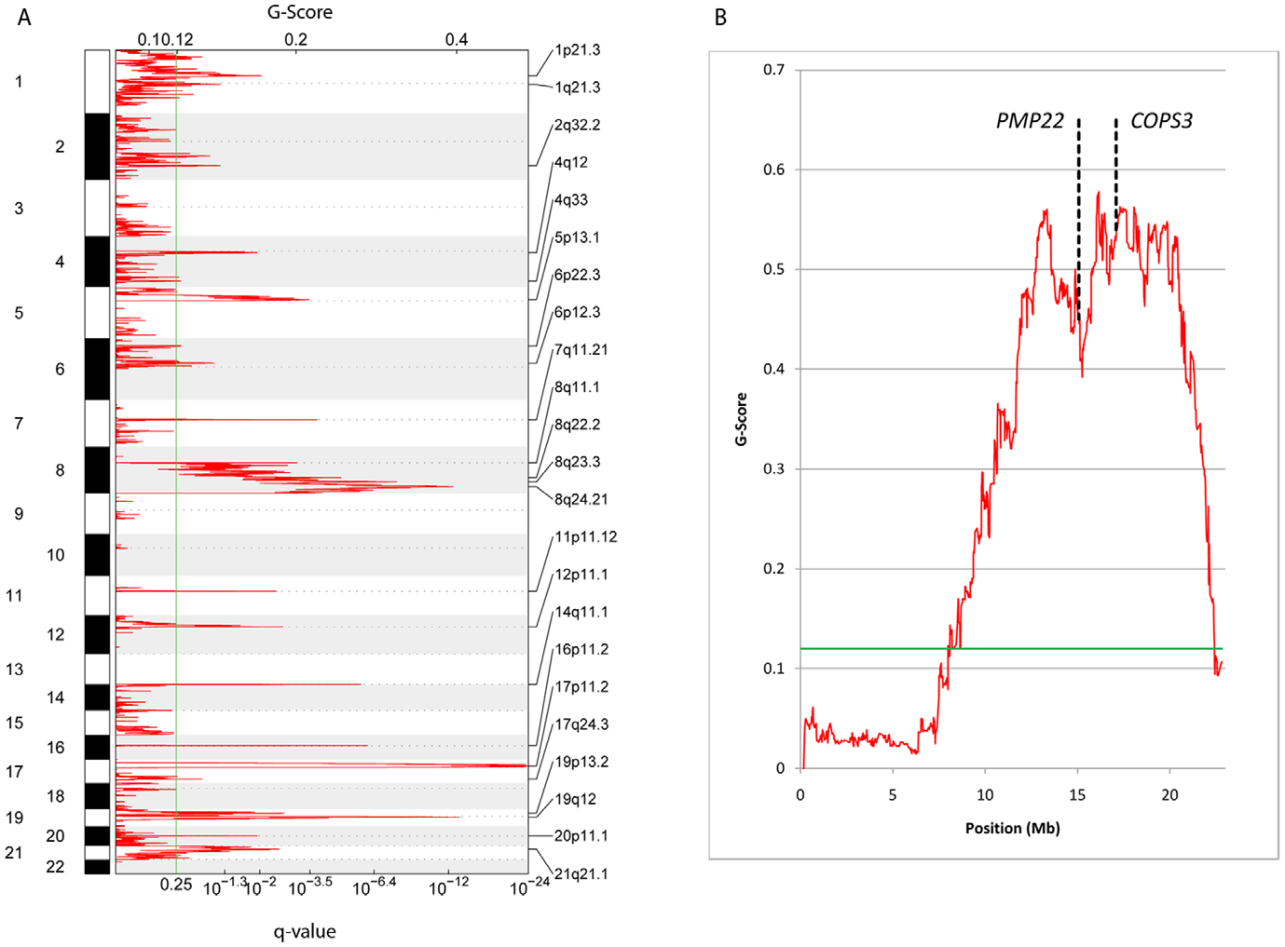
GISTIC amplification analysis. A. Chromosomal regions showing significant amplification by SNP-array profiling, generated by GISTIC analysis. The significance threshold (q-value 0.25) is indicated by the green line. B. Detailed GISTIC analysis of chromosome arm 17p. Positions of previously suggested candidate oncogenes *COPS3* in 17p11.2 and *PMP22* in 17p12 are indicated. The significance threshold (q-value 0.25) is indicated by the green line.

To select for osteosarcoma samples with amplification events in the 17p11.2-p12 region, we performed qPCR for test genes *SCO1* at 10.6 Mb (p12), *MAP2K4* at 11.9 Mb (p12), *MYOCD* at 12.6 Mb (p12), *COX10* at 14.0 Mb (p12), *PMP22* at 15.1 Mb (p12), *NCOR1* at 16.1 Mb (p11.2-p12), *COPS3* at 17.2 Mb (p11.2), and *TOM1L2* at 17.8 Mb (p11.2) on 85 tumours. Twenty-six osteosarcomas displayed amplification events (normalized copy number >3.5) for one or more of the test genes. These samples were subjected to whole genome high-resolution SNP array analysis using Illumina HumanCNV370-Quad BeadChips. The arrays were analyzed with Illumina GenomeStudio software. The resulting LogR ratio (LRR) and the B allele frequency (BAF) data for all SNPs were processed into the OverUnder plugin [22], and absolute copy numbers were calculated using Illumina BeadStudio (see Materials and Methods for details). Amplified regions were found on chromosomes 1, 5–8, 11, 14, 16, 17, 19, and 20 (sex chromosomes excluded). Large deletions were limited to chromosomes 3, 5, 6, 9, 10, and 13. A comparison of the genome-wide copy number profile of these selected tumours with that of unselected osteosarcomas will be presented elsewhere (Both *et al.*, in preparation). Representative chromosome 17 copy number profiles of three osteosarcoma samples (OS2, OS11, OS20) are shown in Figure 1. OS2 exhibits multiple amplification peaks in 17p, superimposed on copy number increases for whole chromosome 17. Figures 1B and 1C show localized amplification events on 17p and diploid copy numbers for the other parts of chromosome 17. The 17p copy number profiles in the other selected osteosarcomas often proved to be complex, as in sample OS2, but always included 17p11.2 and/or 17p12. To determine the most significantly amplified segments in these profiles, we subjected the whole genome copy number data to Genomic Identification of Significant Targets in Cancer (GISTIC) analysis. This method identifies regions that are aberrant (amplified or deleted) more often than would be expected by chance, and gives greater weight to high-amplitude events [24]. The whole genome GISTIC amplification analysis is shown in Figure 2A, a detailed view for chromosome arm 17p is presented in Figure 2B. As expected, the amplification event on chromosome arm 17p (marked by 17p11.2) proved to be most significant, as this was the condition of inclusion. The GISTIC amplification profile also demonstrates significant peaks for chromosomal regions 6p12.3, 8q24.21, and 19q12, containing the recently identified ostosarcoma candidate oncogenes *RUNX* and *CDCL5*, *MYC*, and *CCNE1*, respectively [11]–[13], [25]. The detailed GISTIC analysis for 17p shows a minimal significant amplified segment in the 17p11.2-p12 region, with two major peaks centered on 13.5 and 17.2 Mb, respectively. The positions of *PMP22* and *COPS3*, which we previously suggested as candidate oncogenes in this region, are indicated.

### Integration of copy number and expression data for genes in 17p11.2-p12

Overexpression as a consequence of gene amplification is a major mechanism for oncogene activation in tumours [26], [27]. Thus, the candidate oncogenes in the 17p11.2-p12 region should be found to be overexpressed in a significant fraction of the tumours in which they were amplified. In addition, the amplification-induced overexpression of these genes should result in a positive correlation between copy number and expression level in the respective tumours. To identify these candidate genes, we determined expression levels for all genes in the two established amplicons. Expression data for the genes in this region could be generated for 20 of the 26 osteosarcoma samples that were subjected to SNP array analysis. We extracted the expression data from a whole genome expression profiling analysis of 35 osteosarcoma samples with 17p11.2-p12 amplifications and of human osteoblasts, using Illumina HT12 expression array BeadChips. A comparison of the genome wide expression profile of these selected tumours with that of unselected osteosarcomas will be presented elsewhere (Both *et al.*, in preparation). To establish the correlation between overexpression and amplification, we first calculated for each gene in the 17p11.2-p12 segment in each of the 20 tumours the fold-change in expression relative to the expression in normal osteoblasts. Genes with a two-fold or more increase in normalized expression were considered to be overexpressed. In this way, we identified 53 genes that proved to be overexpressed in at least one of the 20 osteosarcoma samples. We then determined copy numbers for each of these genes in each tumour using the absolute copy numbers of SNPs within or nearest to their coding sequence. The latter were extracted from the preceding SNP array analyses. Genes with copy number >3.5 were considered to be amplified. The amplification and overexpression status of each of the 53 genes in the 20 tumours with 17p11.2-p12 amplification is given in Supplemental Figure S1.

Based on this correlative analysis, we identified the 11 genes listed in Table 1, which proved to be overexpressed as a consequence of amplification in more than 50% of cases.

**Table 1:** Top 11 genes most frequently overexpressed through amplification in 17p11.2-p12.

Gene	Location	Band Mb	A	O	O/A	%
*SHMT1*	p11.2	18.25	17	15	13	76.5
*PMP22*	p12	15.15	16	14	12	75
*RASD1*	p11.2	17.39	18	13	12	66.7
*TOP3A*	p11.2	19.5	17	12	11	64.7
*PRPSAP2*	p11.2	17.8	16	10	10	62.5
*COPS3*	p11.2	17.17	18	12	11	61.1
*GRAP*	p11.2	18.94	17	12	10	58.8
*C17orf39*	p11.2	17.95	18	10	10	55.6
*RICH2*	p12	12.75	17	11	9	52.9
*ALKBH5*	p11.2	18.1	17	10	9	52.9
*C17orf45*	p11.2	16.34	18	10	9	50

Genes were scored for the number of times a feature was found in the tumour (data taken from Supplemental Figure S1). A: number of tumours with amplification of a gene; O: number of tumours with overexpression of that gene; O/A: number of tumours with overexpression and amplification of that gene; %: percentage of tumours in which the amplified gene is overexpressed.

To assess the significance of the association between copy number and expression for these top ranking genes, we performed qPCR and qRT-PCR for each of the genes in each tumour and established the strength of the relationship between these two variables by calculating Pearson's correlation coefficient *R*. Using a positive *R* of approximately 0.5 or more and a 2-sided *P* of 0.05 or less as cut off values for a statistically significant association between copy number and expression, we discarded two of the eleven top genes listed in Table 1, i.e. *GRAP* and *ALKBH5*, as candidate oncogenes in 17p11.2-p12 (Table 2).

**Table 2:** Pearson's correlation coefficient *R* estimating the relationship between gene copy number and expression level for the top 11 genes most frequently overexpressed through amplification in 17p11.2-p12.

Gene	Function of gene product	*R*	2-sided *P*
*C17orf39*	Subunit of complex that co-activates transcription from RNA pol II promoters	0.77	0.00003
*RICH2*	GTPase activator for Rho-type GTPases	0.75	0.00009
*C17orf45*	Non-coding RNA with unknown function	0.74	0.0001
*TOP3A*	Alteration of DNA topologic state during transcription	0.73	0.0001
*COPS3*	Embryonic development/signal transduction	0.72	0.0002
*SHMT1*	One-carbon compound metabolism	0.67	0.0007
*PRPSAP2*	Nucleic acid metabolism	0.64	0.001
*PMP22*	Component of myelin/cell growth control	0.62	0.002
*RASD1*	Activator of G-protein signaling	0.49	0.02
*GRAP*	Cytoplasmic signaling	−0.13	0.6
*ALKBH5*	Alkylation repair homolog	−0.16	0.5

## Discussion

Osteosarcomas are characterized by the presence of highly complex genomic aberrations. The p11.2-p12 region on chromosome 17 has already been known for some time to be amplified in a substantial fraction (25%) of these tumours [9], [10], [15]–[19]. However, the great variation and complexity of the amplification profiles in this region, exemplified in Figure 1, have hampered the identification of the driver oncogenes for these amplifications. In this study, we performed a systematic search for these genes based on the principle that a causative gene should exert its oncogenic action via amplification-induced overexpression. We first selected tumours with17p11.2-p12 amplifications. This enabled us to enrich the dataset for amplification events in our area of interest. Secondly, we applied the GISTIC procedure, which incorporates not only the frequency but also the amplitude of amplifications events, to demarcate the most significantly amplified segments in 17p11.2-p12. The amplification event on chromosome 17 was clearly restricted to the 17p11.2-p12 region and could be subdivided into two major amplicons (Figure 2B). However, the critical region was still quite large (approximately 13 Mb), encompassing 53 genes that exhibited overexpression in at least one of the tumours for which microarray expression data were available. These genes were subsequently scored for amplification and overexpression status, resulting in a top list of 11 genes that were overexpressed through amplification in at least 50% of cases (Table 1). Based on our statistical analysis of the correlation between copy number increase and expression level for each of the top ranking genes, we identified *RICH2*, *c17orf45*, *TOP3A*, *COPS3*, *SHMT1*, *PRPSAP2*, *PMP22*, and *RASD1* as candidate osteosarcoma oncogenes in the 17p11.2-p12 region (Table 2).

The gene list includes *COPS3* in 17p11.2 and *PMP22* in 17p12, which we [14] and others [28], [29] previously suggested to act as causative oncogenes in osteosarcoma tumourigenesis. In a recent study [30], further support for an oncogenic role for *COPS3* was provided by demonstrating that RNAi-mediated *COPS3* gene silencing inhibits the metastatic potential of osteosarcoma cells, suggesting that *COPS3* overexpression might have an important role in the metastasis of osteosarcoma cells. The PMP22 protein is a component of the myelin sheath in the peripheral nervous system [31]. Next to its structural role, PMP22 has important functions in cell growth control in non-neural tissues. We [32] and others [33] have previously reported upregulation of *PMP22* expression in osteosarcoma tumours and cell lines. Since then, increased *PMP22* expression has also been noted in invasive versus non-invasive mammary carcinoma cell lines [34], in (pre)malignant lesions versus normal pancreatic tissue [35], and in proliferative versus secretory phase endometrium [36]. Taken together, these data suggest that, contrary to its original proposed property as being associated with cell growth arrest [37], *PMP22* has an important proliferation-associated and oncogenic role, not only in osteosarcoma tumourigenesis, but in the development of other tumour types as well.

*C17orf39* codes for the hypothetical protein LOC79018, which is thought to be a subunit of the Mediator complex. This complex functions as a coactivator required for activation of RNA polymerase II transcription by DNA bound transcription factors. Although its oncogenic potential is not directly obvious, it is interesting to note that *C17orf39* and also *PRPSAP2* were recently found to be overexpressed in osteosarcoma cell lines [25]. The transcript of *C17orf45* is a non-protein coding RNA with unknown biological function.

The RICH2 protein has a GAP domain that functions as a GTPase activator for Rho-type GTPases [38], [39]. The latter constitute a subfamily of the RAS superfamily of small GTPases. Rab proteins also belong to this family and TBC1D16, encoding Rab3-GAP, has recently been identified as a driver oncogene in melanoma [40]. In analogy, *RICH2* might have a comparable function in osteosarcoma tumourigenesis.

The gene product of *RASD1* is another member of the RAS superfamily of small GTPases and, as such, presumed to have an oncogenic function. However, Vaidyanathan *et al* [41] found that expression of RASD1 in a number of cell lines suppressed cell growth, contrary to what would be expected of RAS family members. This would make *RASD1* a poor candidate oncogene for osteosarcoma tumourigenesis, However, in accordance with an oncogenic function, knockdown of the expression of *RABB27A*, encoding another member of the Ras superfamily, was shown to inhibit the growth of melanoma cells [40]. In analogy, *RASD1* might have such an oncogenic aspect as well.

Two types of topoisomerases are found in mammals, type I which alters topology by a single strand break and type II which work through a double stranded break. We [19] and others [42] have previously reported amplification of *TOP3A*, encoding a type 1 topoisomerase, in osteosarcomas. However no other information about its possible oncogenic potential is available. The implications for TOP2A amplification in cancer are better known. Amplification of *TOP2A*, encoding a type II isomerase, has been reported for a multitude of neoplasms including breast [43], gastric [44], and pulmonary cancers [45], and isolated *TOP2A* amplifications have been found in urinary bladder cancers [46] and acute lymphoblastic leukaemias [47]. In node-negative breast cancer patients high *TOP2A* expression was found to be significantly associated with shorter metastasis-free intervals [48]. Although TOP2A and TOP3A are different types of isomerases, one may speculate that amplification and overexpression of *TOP3A* has an oncogenic effect similar to that of *TOP2A* on the development of osteosarcoma and other cancers.

SHMT1 functions as a regulator in the *de novo* synthesis of thymidine nucleotides. Several polymorphisms in the *SHMT1* gene have been linked to a higher chance of cancer, like acute lymphoblastic leukemia [49], [50], ovarian cancer [51] and prostate cancer [52]. PRPSAP2 is another protein involved in nucleic acid metabolism. The *PRPSAP2* gene encodes part of the enzyme PRPP synthetase, which catalyzes the formation of phosphoribosylpyrophosphate, being a primary substrate for newly formed purine and pyrimidine nucleotides. Depletion of the PRPP synthetase causes growth arrest [53], the reverse has not been shown. Overexpression of *SHMT1* and *PRPSAP2* (and also *COPS3*) has been reported to occur in multiple myelomas [54].

### Conclusion

Our systematic screening of genes in the 17p11.2-p12 region has yielded nine candidate oncogenes associated with osteosarcoma tumourigenesis. Functional studies are underway to test these candidate genes for there oncogenic potential. To test their prognostic potential, we are currently investigating the expression of our candidate genes at the RNA and protein level in an extended series of osteosarcomas with or without metastasis or local recurrence. It has already been demonstrated that there is a significant relationship between strong COPS3 staining and patients that develop metastasis [30]. However, prognostic studies for the other candidate genes require the development of new antibodies (*C17orf39*, *RICH2*, *RASD1*) or testing of available antibodies on osteosarcoma tissues (*TOP3A*, *COPS3*, *SHMT1*, *PRPSAP2*, *PMP22*), which is presently underway. The candidate oncogenes could be useful as biomarkers for improvement of diagnosis and as possible targets for therapeutic intervention in osteosarcoma and in other tumour types as well.

## Supporting Information

Figure S1 Amplification and overexpression status of genes in chromosome region 17p11.2-p12 in osteoarcoma. Cells are colored as follows. Blue: gene amplified, yellow: gene overexpressed, green: gene amplified and overexpressed, blank: gene without amplification and overexpression.
(TIF)

Table S1 qPCR primers.
(DOC)

Table S2 qRT-PCR Primers.
(DOC)
