# Supplementary figures and images for: Identification of Novel Candidate Oncogenes in Chromosome Region 17p11.2-p12 in Human Osteosarcoma
Source: PLoS One. 2012 Jan 26;7(1):e30907. doi: 10.1371/journal.pone.0030907 (PMC3266911; doi:10.1371/journal.pone.0030907)

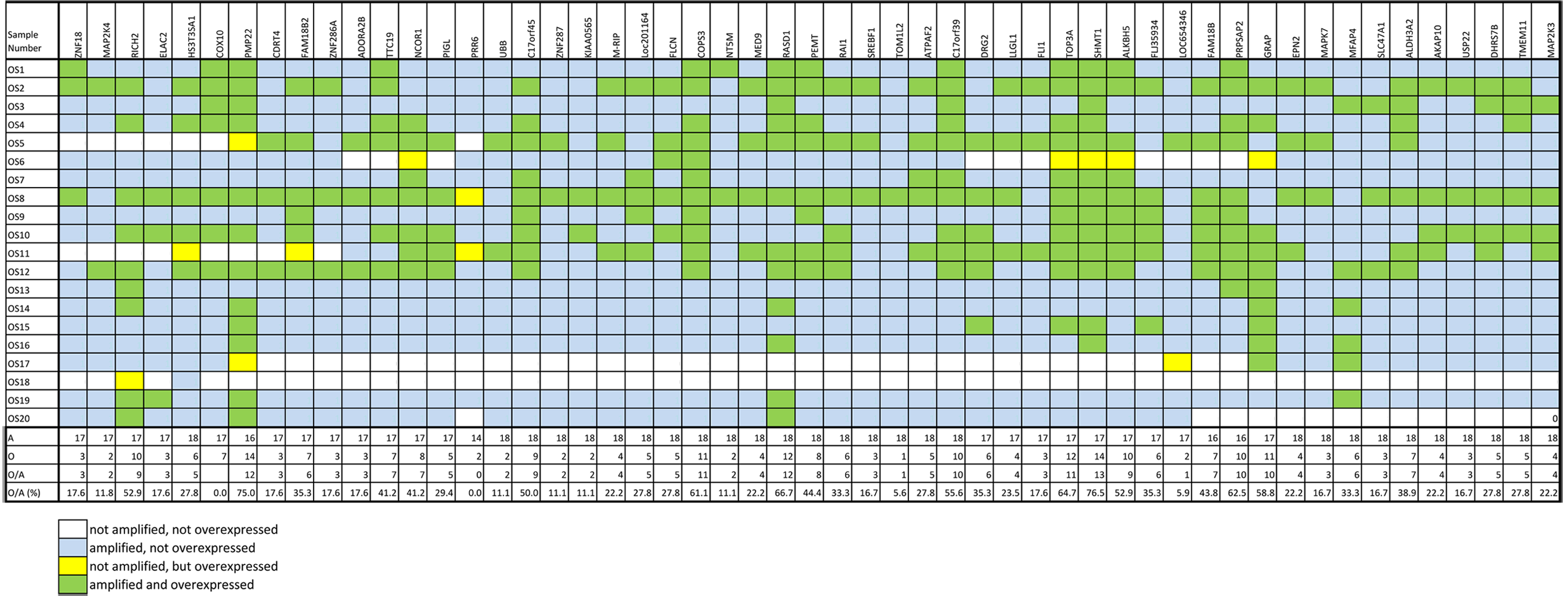

Supplement: Figure S1 — Amplification and overexpression status of genes in chromosome region 17p11.2-p12 in osteoarcoma. Cells are colored as follows. Blue: gene amplified, yellow: gene overexpressed, green: gene amplified and overexpressed, blank: gene without amplification and overexpression. (TIF) [file pone.0030907.s001.tif]
