# Supplementary material for: Identification of Novel Candidate Oncogenes in Chromosome Region 17p11.2-p12 in Human Osteosarcoma
Source: PLoS One. 2012 Jan 26;7(1):e30907. doi: 10.1371/journal.pone.0030907 (PMC3266911; doi:10.1371/journal.pone.0030907)
Supplement: Table S1 — qPCR primers. (DOC) [file pone.0030907.s002.doc]

Table S1

| Gene  (ID) | Primer Name | Primer Length (b) | Primer Sequence | Probe nr/SG |
| --- | --- | --- | --- | --- |
| ALB | Gno_ALB_II_F | 19 | aatgttgccaagctgctga | 27 |
| (213) | Gno_ALB_II_R | 20 | cttcccttcatcccgaagtt |  |
| SCO1 | Gno_SCO1_F | 20 | gtcccttcctttgcatcact | 18 |
| (6341) | Gno_SCO1_R | 26 | gcaaagatttcctaatttaattgtca |  |
| MAP2K4 | Gno_MAP2K4_F | 27 | ctggaaataattgaagacaaaattagg | 17 |
| (6416) | Gno_MAP2K4_R | 23 | ttcatataatctgtgccatgctg |  |
| MYOCD | Gno_MYOCD_F | 20 | gactcgccgatgatctcaat | 31 |
| (93649) | Gno_MYOCD_R | 20 | tcacagcagaatccacagga |  |
| COX10 | Gno_COX10_F | 20 | ggggctcttcaacattttcc | 62 |
| (1352) | Gno_COX10_R | 20 | gttggcaatgctgatccttt |  |
| PMP22 | Gno_PMP22_II_F | 20 | tccttgggatagccagtacc | 18 |
| (5376) | Gno_PMP22_II_R | 25 | tgttaaagaacttaaagccgaaaca |  |
| NCOR1 | Gno_NCOR1_F | 20 | attcggaggcaaacatgaag | 55 |
| (9611) | Gno_NCOR1_R | 23 | aagattttgatcatctccacat |  |
| COPS3 | Gno_COPS3_F | 23 | tgctgtagctgaaatgcaatagt | 37 |
| (8533) | Gno_COPS3_R | 23 | ttgttgatcagttcacaaagctg |  |
| TOM1L2 | Gno_TOM1L2_F | 21 | tcgaagcagtcctgatctcac | 17 |
| (146691) | Gno_TOM1L2_R | 19 | tgggaaattcaaccccttt |  |
| PRPSAP2 | Gno_PRPSAP2_F | 21 | tggagatccccagtaagtgtg | 34 |
| (5636) | Gno_PRPSAP2_R | 24 | cagccttaaaatcagaggaaagtg |  |
| ALKBH5 | Gno_ALKBH5_F | 19 | tctaagggcctcacggaag | 44 |
| (54890) | Gno_ALKBH5_R | 21 | tctgtccaaccagaaagcact |  |
| RICH2 | Gno_RICH_F | 20 | ccttccacccatctcaagtc | 66 |
| (9912) | Gno_RICH_R | 20 | ggcccccagaatcctagtaa |  |
| c17orf39 | Gno_c17orf39_F | 21 | ctttcaccactcatggaggac | 1 |
| (79018) | Gno_c17orf39_R | 20 | acaaacccaggtgcagtaca |  |
| c17orf45 | Gno_c17orf45_F | 19 | gcttgtgacaggatggttt | 4 |
| (125144) | Gno_c17orf45_R | 23 | aggagcagtaaaaacaacgtcag |  |
| GRAP | Gno_GRAP_IV_F | 20 | cttgagcttgggctctgcag | SG |
| (10750) | Gno_GRAP_IV_R | 23 | gagggaacactgaggggat |  |
| RASD1 | Gno_RASD1_F | 19 | aggacttccaccgcaagtt | 58 |
| (51655) | Gno_RASD1_R | 20 | cgaggatgtcgagctggtag |  |
| TOP3A | Gno_TOP3A_F | 19 | gacagtgaggggaggggta | 19 |
| (7156) | Gno_TOP3A_R | 18 | gccagccctttccctaat |  |
| SHMT1 | Gno_SHMT1_F | 19 | tcctgtgggggttcagatt | 77 |
| (6470) | Gno_SHMT1_R | 20 | caaggatcccaggtcagagt |  |

Table S1. qPCR primers, SG: SybrGreen labeling.
