# Supplementary material for: Identification of Novel Candidate Oncogenes in Chromosome Region 17p11.2-p12 in Human Osteosarcoma
Source: PLoS One. 2012 Jan 26;7(1):e30907. doi: 10.1371/journal.pone.0030907 (PMC3266911; doi:10.1371/journal.pone.0030907)
Supplement: Table S2 — qRT-PCR Primers. (DOC) [file pone.0030907.s003.doc]

Table S2

| Gene  (ID) | Primer Name | Primer  Length (b) | Primer Sequence | Probe nr/SG |
| --- | --- | --- | --- | --- |
| PMP22 | rt_PMP22_F | 22 | ctgtcgatcatcttcagcattc | 29 |
| (5376) | rt_PMP22_R | 20 | agcactcatcacgcacagac |  |
| COPS3 | rt_COPS3_F | 19 | cccagccatgcttcataac | 42 |
| (8533) | rt_COPS3_R | 18 | tggtccatggctttcagc |  |
| TOP3A | rt_TOP3A_F | 18 | accgtggagatggtggac | 54 |
| (7156) | rt_TOP3A_R | 19 | catgagtggcatccgtacc |  |
| RASD1 | rt_RASD1_F | 20 | ggtctaccagctcgacatcc | 58 |
| (51655) | rt_RASD1_R | 21 | gaacaccaggatgaaaacgtc |  |
| SHMT1 | rt_SHMT1_F | 19 | ccctccccatttgaacact | 77 |
| (6470) | rt_SHMT1_R | 21 | gggatccacacttttcactcc |  |
| PRPSAP2 | rt_PRPSAP2_F | 21 | ggattgaagagtctgccattg | 77 |
| (5636) | rt_PRPSAP2_R | 21 | agcttctggacttcatgtgga |  |
| ALKBH5 | rt_ALKBH5_F | 19 | agggaccctgctctgaaac | 44 |
| (54890) | rt_ALKBH5_R | 20 | tccttgtccatctccaggat |  |
| RICH2 | rt_RICH2_F | 20 | cctggggaaagcatgtctac | 1 |
| (9912) | rt_RICH_R | 20 | ccgagtccctcttgtcagtt |  |
| c17orf39* | rt_c17orf39_F | 20 | ctctacagcggctccaagtt | 39 |
| (79018) | rt_c17orf39_R | 20 | gtaagagttccccgtgtcca |  |
| c17orf45* | rt_c17orf45_F | 20 | gctgagtattggagccagga | 74 |
| (125144) | rt_c17orf45_R | 20 | ccagctcaaggcttaccttc |  |
| GRAP | rt_GRAP_Veg_F | 21 | tggagggaggagcccttggag | SG |
| (10750) | rt_GRAP_Veg_R | 21 | aatacacaggctgggtcagga |  |
| SDHA | rt_SDHA_F | 19 | agaagccctttgaggagca | 69 |
| (6389) | rt_SDHA_R | 21 | cgattacgggtctatattcca |  |

Table S2. qRT-PCR Primers. cDNA was made with oligo-dT or random(*) priming. SG: SybrGreen labeling
